# Short-Term Fever-Range Hyperthermia Accelerates NETosis and Reduces Pro-inflammatory Cytokine Secretion by Human Neutrophils

**DOI:** 10.3389/fimmu.2019.02374

**Published:** 2019-10-18

**Authors:** Irene A. Keitelman, Florencia Sabbione, Carolina M. Shiromizu, Constanza Giai, Federico Fuentes, David Rosso, Camila Ledo, Maximiliano Miglio Rodriguez, Mauricio Guzman, Jorge R. Geffner, Jeremías Galletti, Carolina Jancic, Marisa I. Gómez, Analía S. Trevani

**Affiliations:** ^1^Laboratorio de Inmunidad Innata, Instituto de Medicina Experimental (IMEX)-CONICET, Academia Nacional de Medicina, Buenos Aires, Argentina; ^2^Instituto de Investigaciones en Microbiología y Parasitología Médica (IMPaM) UBA-CONICET, Buenos Aires, Argentina; ^3^Laboratorio de Microscopía, Instituto de Medicina Experimental (IMEX)-CONICET, Academia Nacional de Medicina, Buenos Aires, Argentina; ^4^Departamento de Investigaciones Biomédicas y Biotecnológicas, Centro de Estudios Biomédicos, Biotecnológicos, Ambientales y de Diagnóstico (CEBBAD), Universidad Maimónides, Buenos Aires, Argentina; ^5^Consejo Nacional de Investigaciones Científicas y Técnicas (CONICET), Buenos Aires, Argentina; ^6^Instituto de Investigaciones Biomédicas en Retrovirus y SIDA (INBIRS), CONICET, Facultad de Medicina, Universidad de Buenos Aires, Buenos Aires, Argentina; ^7^Departamento de Microbiología, Parasitología e Inmunología, Facultad de Medicina, Universidad de Buenos Aires, Buenos Aires, Argentina

**Keywords:** neutrophils, hyperthermia, NETosis, NETs, cytokines

## Abstract

Fever is a hallmark of infections and inflammatory diseases, represented by an increase of 1–4°C in core body temperature. Fever-range hyperthermia (FRH) has been shown to increase neutrophil recruitment to local sites of infection. Here, we evaluated the impact of a short period (1 h) of FRH (STFRH) on pro-inflammatory and bactericidal human neutrophil functions. STFRH did not affect neutrophil spontaneous apoptosis but reverted the lipopolysaccharide (LPS)-induced anti-apoptotic effect compared with that under normothermic conditions. Furthermore, STFRH accelerated phorbol myristate acetate (PMA)-induced NETosis evaluated either by the nuclear DNA decondensation at 2 h post-stimulation or by the increase in extracellular DNA that colocalized with myeloperoxidase (MPO) at 4 h post-stimulation. Increased NETosis upon STFRH was associated with an increase in reactive oxygen species (ROS) production but not in autophagy levels. STFRH also increased NETosis in response to *Pseudomonas aeruginosa* challenge but moderately reduced its phagocytosis. However, these STFRH-induced effects did not influence the ability of neutrophils to kill bacteria after 4 h of co-culture. STFRH also significantly reduced neutrophil capacity to release the pro-inflammatory cytokines chemokine (C-X-C motif) ligand 8/interleukin 8 (CXCL8/IL-8) and IL-1β in response to LPS and *P. aeruginosa* challenge. Altogether, these results indicate that a short and mild hyperthermal period is enough to modulate neutrophil responses to bacterial encounter. They also suggest that fever spikes during bacterial infections might lead neutrophils to trigger an emergency response promoting neutrophil extracellular trap (NET) formation to ensnare bacteria in order to wall off the infection and to reduce their release of pro-inflammatory cytokines in order to limit the inflammatory response.

## Introduction

Neutrophils are the most numerous leukocytes in human circulation. These cells are rapidly and massively recruited to tissues in response to multiple microbial and sterile challenges (1). Their localization to the site of inflammation is essential for the clearance of infections (2). When arriving at the site of infection, they recognize the insulting pathogens and deploy an extensive repertoire of antimicrobial weapons that includes oxidants, proteases, and antimicrobial proteins (3). Neutrophils mediate the killing of microbes through phagocytosis, degranulation, and neutrophil extracellular trap (NET) generation (3).

NETs are fibrous structures that usually consist of a nuclear DNA scaffold with associated histones and nuclear, cytoplasmic, and granular proteins. They are released by neutrophils in response to microbial and sterile agents and play an important role in the defense against bacteria, fungi, and viruses (4–6). NETs ensnare microorganisms to prevent their dissemination and wall off the infection (7). Their associated antimicrobial compounds, such as proteases, antimicrobial peptides, and histones, can directly kill pathogens (6).

Although neutrophils play an indisputable role as microbicidal cells, evidence from recent years indicates that their biological functions transcend the killing of pathogens (8). In fact, human neutrophils produce potent pro-inflammatory cytokines and chemokines like chemokine (C-X-C motif) ligand 8/interleukin 8 (CXCL8/IL-8), interleukin 1 beta (IL-1β), and tumor necrosis factor alpha (TNF-α). The huge daily generation of neutrophils (~10^11^), which dramatically increases upon infection/inflammation (9), together with their massive recruitment to inflammatory foci suggests that neutrophil-derived cytokines might be of foremost importance.

Fever is a cardinal response to infection. Given that it has been conserved throughout evolution, it is thought that it confers a survival advantage. Indeed, substantial evidence has accumulated suggesting that febrile temperatures are associated with improvement in survival and the resolution of infections (10). Previous studies showed that fever-range hyperthermia (FRH) increases neutrophil pulmonary recruitment in experimental Gram-negative bacterial pneumonia (11). Even though an augmented expression of CXC chemokines was involved in the hyperthermia-mediated enhancement of neutrophil recruitment to the lungs (12), additional evidence indicated that FRH also exerts direct effects on both neutrophils and the endothelium that augment their extravasation (13). Other studies showed that both spontaneous and lipopolysaccharide (LPS)-induced reactive oxygen species (ROS) production by adherent human neutrophils are enhanced at febrile temperatures (14). Earlier studies found contrasting findings regarding the impact of hyperthermia on the neutrophil capacity to kill pneumococci and *Escherichia coli in vitro* (15). Besides, a slight although significant increase in neutrophil bactericidal capacity against *E. coli, Salmonella typhimurium*, and *Listeria monocytogenes* at 40°C was reported to ensue at 1 h but was not detectable at 2 h. This effect was not observed with *Staphylococcus aureus* (16).

In this study, we evaluated the impact of a short period (1 h) of FRH (STFRH; 39.5°C) on microbicidal and pro-inflammatory functions of human neutrophils and on its capacity to fight against *Pseudomonas aeruginosa* infections.

## Materials and Methods

The experimental protocols performed were approved by the Biosafety and Research Review Board of IMEX-CONICET-ANM and the Ethical Committee of the Institutos de la Academia Nacional de Medicina. The methods were carried out in accordance with the approved guidelines.

### Reagents and Materials

Roswell Park Memorial Institute (RPMI) 1640 culture media, Pierce lactate dehydrogenase (LDH) Cytotoxicity Assay Kit, TO-PRO-3, and TMB substrate were purchased from Thermo Fisher Scientific (Massachusetts, MA, USA). Fetal bovine serum (FBS) was purchased from Internegocios (Buenos Aires, Argentina). Luria broth (LB) medium was purchased from Acumedia (Michigan, USA), bacteriological agar was purchased from Britania (Buenos Aires, Argentina). Ficoll was purchased from GE Healthcare (Munich, Germany). DNase (Dornasae alpha) was from Roche, Argentina. Anti-myeloperoxidase (MPO)–fluorescein isothiocyanate (FITC) antibody was purchased from Biolegend (San Diego, USA); rabbit gamma globulin, anti-rabbit Alexa 647, and Alexa Fluor 488 F(ab′)_2_ fragment goat anti-rabbit IgG cat. #111-546-144 were purchased from Jackson ImmunoResearch Laboratories (West Grove, PA, USA). Rabbit polyclonal antibody anti-LC3B cat. #sc28266 was from Santa Cruz Biotechnology (Dallas, TX, USA). SYBR Gold and Sytox Green were from Life Technologies (Carlsbad, CA, USA). Phycoerythrin-conjugated anti-CD14 antibody; the OptEIA human IL-1β, CXCL8/IL-8, and TNF-α enzyme-linked immunosorbent assay (ELISA) sets; and substrate reagents A and B were purchased from BD Biosciences (Franklin Lakes, NJ, USA). Aqua-Poly/Mount coverslipping medium was purchased from Polysciences (Warrington, PA, USA). Lab-Tek chambers were purchased from Nalge Nunc International, New York, NY, USA. NucSpot live 488 was from Biotium (Fremont, CA, USA). Anti-green fluorescent protein (GFP) antibody was purchased from GenScript (Piscataway, NJ, USA). Unless otherwise stated, all the chemicals employed were from Sigma-Aldrich (Merck KGaA, Darmstadt, Germany).

### Human Neutrophil Isolation

Neutrophils were isolated from heparinized human blood from healthy donors who gave written informed consent, by centrifugation on Ficoll-Paque, dextran sedimentation, and hypotonic lysis (17). Cells were suspended at 5 × 10^6^/mL in RPMI 1640 supplemented with 10% FBS previously heated at 65°C for 30 min for nuclease inactivation, and with or without penicillin (100 U/mL) and Streptomycin (100 μg/mL). After isolation, neutrophil preparations were stained with an anti-CD14-PE antibody and analyzed with a FACSCalibur (Beckton Dickinson, San Jose, CA, USA) or a CyFlow cytometer (Sysmex Partec, Germany) to guarantee that monocyte contamination was <0.5%. Cells were used immediately after isolation.

### Bacterial Strains

*Pseudomonas aeruginosa* PAO-1 strain was kindly provided by Prof. Barbara Iglewski (Department of Microbiology and Immunology, University of Rochester, Rochester, NY). GFP-tagged PAO-1 strain was kindly provided by Prof. Tim Tolker-Nielsen (Centre for BioScience and Technology, Technical University of Denmark, Lyngby, Denmark).

*P. aeruginosa* was grown on Luria broth agar (LA) plates and kept at 4°C. For individual experiments, the organism was grown overnight in LB medium at 37°C and then was diluted in fresh LB medium and grown at 37°C with agitation until an OD600 of 0.6. After that, it was washed twice by centrifugation and suspended in RPMI medium without phenol red. The desired concentration was attained by monitoring spectrophotometric absorbance at 600 nm, using an appropriate optical density/colony-forming units (CFUs) curve.

### Neutrophil Stimulation

Except otherwise stated, neutrophils were cultured for 1 h at 37 or 39.5°C in the presence or absence of phorbol myristate acetate (PMA) (25 ng/mL) or LPS from *E. coli* O111:B4 (250 ng/mL) and then were cultured for one or three additional hours at 37°C (PMA) or 4 h (LPS). In some experiments, 2 h after stimulation with LPS, cells were treated with ATP (2.5 mM). In another set of experiments, neutrophils were first cultured for 1 h at 37 or 39.5°C and then were challenged with *P. aeruginosa* PAO-1 and cultured for three or four additional hours. When indicated, Ac-YVAD-cmk (50 μM) was added 20 min before PMA stimulation. After culture, supernatants were collected and employed to measure DNA or MPO, or were frozen at −20°C until cytokines concentrations were determined by ELISA, following the manufacturer's instructions. In cell pellets, viability was determined by annexin V-FITC/propidium iodide (PI) or VivaFix dye staining and flow cytometry analysis.

### Microscopic Assessment of NET Formation

At the end of the experiment, neutrophils were fixed with 4% paraformaldehyde (PFA), permeabilized with acetone in PBS for 7 min, rehydrated for 7 min, and blocked with 5% goat serum for 60 min at 37°C. Then, they were incubated with a FITC-conjugated anti-MPO antibody, or the corresponding isotype control for 1 h. Then DNA was stained with PI (1 μg/mL) for 10 min, and cells were mounted using Aqua-Poly/Mount coverslip medium. Images were acquired by using a FluoView FV1000 confocal microscope (Olympus, Tokyo, Japan) equipped with a Plapon 60 × /NA1.42 objective and then analyzed with FIJI software.

### NET Quantification

NETs were quantified by determining the concentrations of DNA and MPO in culture supernatants. DNA was quantified by SYBR Gold or Sytox Green (1:2,000) staining and fluorometry detection after interpolation from a standard DNA concentration curve as previously described (18). MPO concentration was quantified by reaction of supernatants containing NETs with BD substrate reagent (A + B) for 10 min. The change in optical density at 450–570 nm was measured by spectrophotometry, and concentration was calculated by interpolation from a standard MPO curve. Alternatively, NETosis was evaluated by determining the number of cells with nuclear DNA decondensation after fixing with 4% PFA, PI staining, and confocal laser scanning microscopy (CLSM).

### LDH Assay

After culture in the presence or absence of PAO-1 (multiplicity of infection (MOI) 0.1), supernatants were harvested and LDH levels were measured by using a Pierce LDH Cytotoxicity Assay Kit. The same number of cells was lysed with 0.5% Triton X-100 and used as a positive control.

### Intracellular LC3 Immunostaining, CLSM Acquisition, and Automated Image Analysis

At the end of the experiment, after fixation with PFA 4% for 30 min, cells were blocked with PBS–glycine (0.1 M) for 15 min, permeabilized with chilled acetone (−20°C) for 7 min, rehydrated with PBS, and blocked with PBS supplemented with 5% goat serum overnight at 4°C. Then, neutrophils were incubated with an anti-LC3B antibody for 1 h at room temperature, washed, and then incubated with the corresponding secondary Alexa 488-conjugated antibody for 1 h at room temperature. Then, PI was added for nuclei staining for 10 min. Afterwards, cells were washed, mounted with Aqua-Poly/Mount mounting medium, and stored at 4°C until confocal microscopy examination. Images were analyzed, and the fluorescence was quantitated with a specific macro with Fiji software (National Institutes of Health, NIH) as we previously described (17).

### Dihydrorhodamine 123 (DHR123) Flow Cytometry Assay

Neutrophils were loaded with DHR123 (10 μM) for 5 min at 37°C. Then, they were cultured for 1 h at 37 or 39.5°C and 15 min before the end of the experiment and were stimulated or not with PMA (25 ng/mL). Then, fluorescence was determined by flow cytometry.

### Phagocytosis Assay

Phagocytosis assays were conducted as previously described with minor modifications (19). Briefly, neutrophils were cultured for 1 h at 37 or 39.5°C and then were incubated for 10 min at 37°C in the presence or absence of NaN_3_ (100 μM). Then GFP-tagged *P. aeruginosa* PAO-1 (MOI 10) or vehicle was added, and cells were co-cultured with shaking for 15 min at 37°C. Afterwards, non-ingested bacteria were removed from neutrophils by centrifugation, and media were replaced. Neutrophils were incubated at 37°C for an additional 45 min. After fixation with PFA 4%, neutrophil fluorescence was determined by flow cytometry. In some experiments, cells were immediately stained at 4°C with an anti-GFP rabbit antibody followed by an Alexa 647 anti-rabbit secondary antibody. Alternatively, cells were cytospinned, mounted with Aqua-Poly/Mount mounting medium, and examined by CLSM.

### Killing Assay

*P. aeruginosa* (PAO-1) was cultured for 2 h in LB medium and then was washed and suspended in RPMI medium without phenol red. Neutrophils were cultured for 1 h at 37 or 39.5°C and then were challenged with PAO-1 at MOI 1 or 0.1 and co-cultured for four additional hours at 37°C and 5% of CO_2_. Then, DNase (10 U/mL) was added for 15 min to release entrapped bacteria. Samples were centrifuged, the supernatants were collected, and the cell pellets were treated with distilled water for neutrophil lysis and mixed with the supernatant fractions. These preparations were plated in duplicates in LB medium. Plates were incubated at 37°C for 18 h, and then the number of CFUs was counted.

### Statistical Analysis

Statistical analysis was performed using GraphPad Prism v6.00 for Windows, GraphPad Software, La Jolla, CA, USA. Comparisons between groups were performed by two-way analysis of variance (ANOVA) with repeated measures followed by Bonferroni's multiple-comparisons test. Otherwise, the Mann–Whitney *U*-test was used for the analysis of two unpaired groups of confocal images. Statistical significance was defined as *p* < 0.05.

## Results

Previous studies have evaluated the effects of hyperthermia on neutrophil functions; however, these works mainly analyzed the impact of prolonged periods of high temperatures (3 h or longer), and in most cases, temperatures evaluated were outside the physiological range. Thus, this study was aimed to determine the effects of a short-term (1 h) FRH on neutrophil functions, to mimic the impact of fever spikes that could take place during infections.

**Figure 1:**
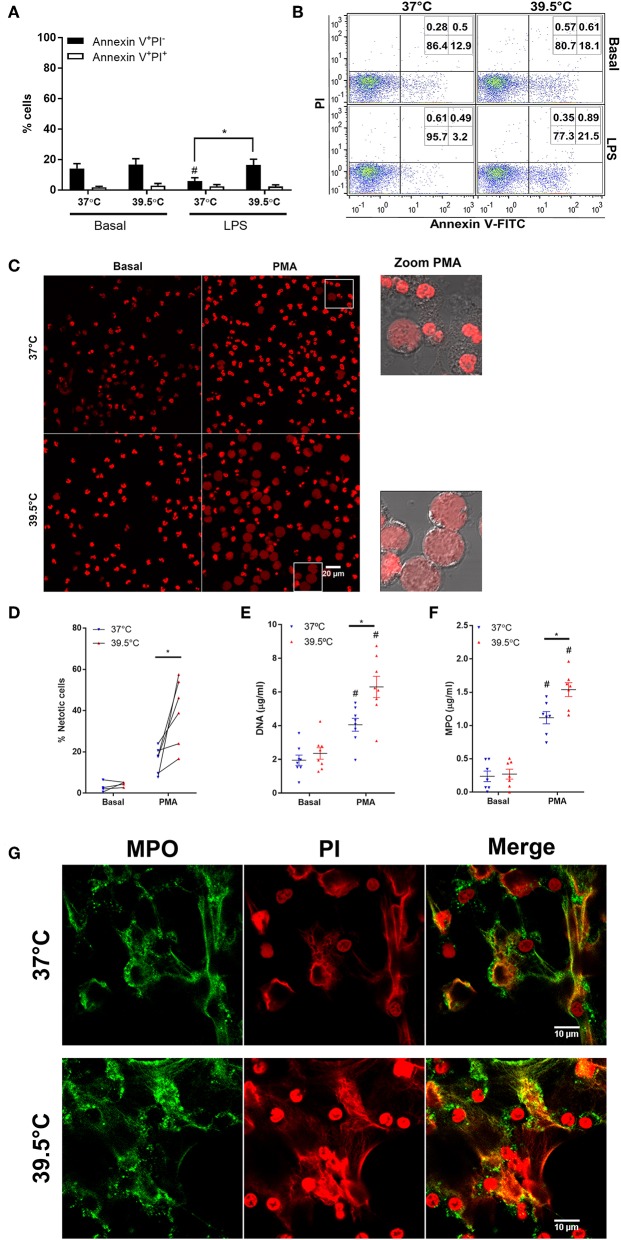
Short-term fever-range hyperthermia accelerates PMA-induced NETosis. Neutrophils were cultured for 1 h at 37 or 39.5°C in the absence (basal) or presence of LPS (250 ng/mL) and then cultured at 37°C for four additional hours **(A,B)** or were cultured for 1 h at 37 or 39.5°C in the absence (basal) or presence of PMA (25 ng/mL) and then cultured at 37°C for one additional hour **(C,D)** or three additional hours **(E–G)**. **(A,B)** After culture, the percentages of apoptotic (annexin V^+^PI^−^) and necrotic (annexin V^+^PI^+^) cells were then evaluated by annexin-V FITC/propidium iodide staining and flow cytometry. **(A)** Data are depicted as the mean ± SEM of four experiments. **(B)** A representative experiment is shown. **(C,D)** Alternatively, after culture, cells were stained with PI and visualized by confocal microscopy. **(C)** Left and center panels, PI fluorescence; zoomed PMA panels, DIC, and PI merge of cells inside the square gate in PMA panels. **(D)** Quantification of cells undergoing NETosis distinguished by their expanded nuclear areas in experiments depicted in **(C)**. At least 300 cells were scored for each treatment per donor. Data correspond to the percentage of cells with expanded nuclear areas like those depicted in zoomed areas in C per donor; individual donors are linked by a connecting line. **(E)** DNA and **(F)** MPO concentrations in culture supernatants collected at 4 h post-stimulation. Bars represent the mean ± SEM of the independent experiments depicted. **(E)** Data are depicted as the mean DNA value of assays performed in triplicate per donor. **(G)** Representative confocal microscopy images of NETs of eight donors identified by MPO and PI staining at 4 h post-stimulation. *p < 0.01 PMA 37°C vs. PMA 39.5°C and ^#^*p* < 0.05 PMA vs. basal at their respective temperatures; two-way ANOVA with Bonferroni's multiple-comparisons test. PMA, phorbol myristate acetate; LPS, lipopolysaccharide; PI, propidium iodide; DIC, differential interference contrast; NETs, neutrophil extracellular traps; MPO, myeloperoxidase; ANOVA, analysis of variance.

In previous studies, we and others determined that prolonged febrile range hyperthermia (8 h) accelerated neutrophil apoptosis (20, 21). By contrast, a shorter period of hyperthermia (1 h) did not affect spontaneous apoptosis and slightly reverted the anti-apoptotic effect of LPS treatment after 20 h of culture (22). Thus, to determine whether STFRH affects apoptosis rates at early time points, we first evaluated its impact on cells that were stimulated or not with LPS for 1 h at 37 or 39.5°C and then cultured for 4 h at 37°C. Results indicated that STFRH did not significantly modulate neutrophil spontaneous apoptosis. However, similarly to what has been previously observed after 20 h, STFRH prevented the LPS-anti-apoptotic effect after the 5 h of culture (Figures 1A,B).

Then we analyzed the impact of STFRH on NETosis induced by PMA. NET formation was first evaluated by analyzing the nuclear expansion that characterizes PMA-induced NETosis. To this end, neutrophils were stimulated or not with 25 ng/mL PMA for 1 h at 37 or at 39.5°C and then cultured for one additional hour at 37°C. Afterwards, cells were stained with PI and analyzed by confocal microscopy. As shown in Figure 1C, and as was confirmed by their quantitation (Figure 1D), STFRH markedly increased the number of cells exhibiting morphologic features of cells undergoing NETosis characterized by nuclear DNA decondensation. This effect was also evident when cells were stimulated with 100 nM (62 ng/mL) of PMA (Figure S1). At this concentration after 2 h post-stimulation (p.s.), some neutrophils have already released NETs when exposed to 39.5°C, but only an incipient NETosis was observed at 37°C. The enhancement effects of STFRH on PMA-induced NETosis were also detected when it was evaluated by DNA and MPO quantitation in culture supernatants of neutrophils that were stimulated with PMA for 1 h at 37 or 39.5°C and then for three additional hours at 37°C (Figures 1E,F). We confirmed that DNA found in supernatants corresponded to NETs and not to DNA released by other cell death pathways, by detection of colocalization of MPO and DNA by confocal microscopy (Figure 1G).

Additional evidence that STFRH was promoting a NETosis program was obtained by differential staining with NucSpot live 488, a cell-permeable DNA dye, and PI, which only penetrates cells with a compromised integrity of their plasma membranes, and by time-lapse live microscopy. As shown in Video S1, since the beginning of the culture, the whole population of neutrophils was stained with NucSpot live 488 (green fluorescence) but not with PI, and it was characterized by brightly condensed lobulated nuclei. As time went on and plasma membrane disintegrated, DNA was released and brightly stained with the extracellular PI, fluorescing yellow by the overlapping of green and red fluorescence signals, which is in agreement with the characteristic steps reported for neutrophils undergoing NETosis under normothermic conditions (5, 23). As shown in Figure S2, nuclei of unstimulated neutrophils subjected to STFRH mainly remained morphologically lobulated at the end of the experiment (3 h 40 min) sharply contrasting with the appearance of PMA-stimulated cells subjected to STFRH.

Additionally, the caspase-1 inhibitor Ac-YVAD-cmk did not significantly modulate DNA and MPO concentrations in culture supernatants regarded as NETosis parameters, at either 37 or 39.5°C (Figure S3). These results further support that hyperthermia promotes a canonical NETosis program, which, as former studies indicated, is a caspase-1-independent process (24).

**Figure 2:**
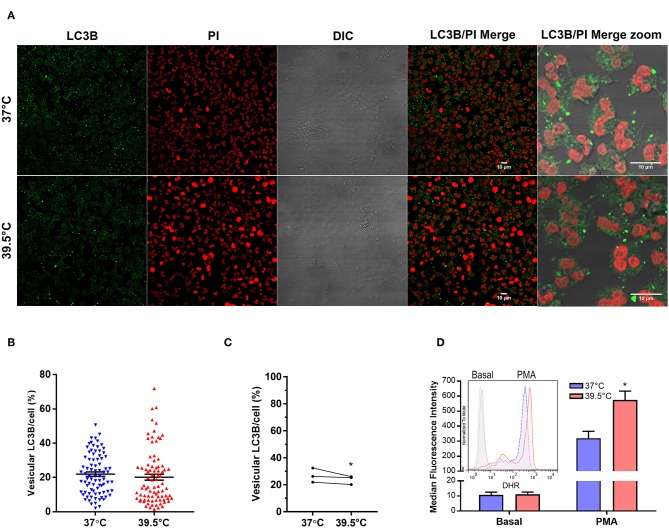
Short-term fever-range hyperthermia increases PMA-induced ROS production but not autophagy levels. **(A)** Representative confocal microscopy images of PMA-stimulated neutrophils cultured for 1 h at 37 or 39.5°C and then stained with a specific antibody anti-LC3B and PI. Image quantifications **(B,C)** were performed by using a specific macro with Fiji software. **(B)** Scatter plot depicts the percentage of vesicular LC3B intensity/cell from a representative experiment of three. Black bars indicate the mean ± SEM values of 83 (37°C) and 84 (39.5°C) cells analyzed. **(C)** Data represent the mean value of vesicular LC3B intensity/cell from experiments like that depicted in **(B)**, performed with three different donors in which at least 69 cells were analyzed for each temperature. *p < 0.05; Mann–Whitney *U*-test analysis. **(D)** Effect of STFRH on neutrophil ROS production induced by PMA. Neutrophils were stained with DHR for 5 min at 37°C and then were split off and stimulated or not with PMA (25 ng/mL) and incubated for 1 h at 37 or 39.5°C, and fluorescence was determined by flow cytometry. Bar graph depicts the median fluorescence intensity of six experiments. Inset shows the histograms of a representative experiment. *p < 0.01 vs. 37°C; two-way ANOVA with Bonferroni's multiple-comparisons test. PMA, phorbol myristate acetate; ROS, reactive oxygen species; STFRH, short period (1 h) of fever-range hyperthermia; DHR, dihydrorhodamine; ANOVA, analysis of variance; PI, propidium iodide.

Previous studies showed that autophagy and ROS production are required for PMA-induced NET formation in human neutrophils (23). Therefore, to obtain insights into the mechanism that leads to NETosis acceleration by STFRH, we then evaluated autophagy levels in neutrophils that were stimulated with PMA for 1 h at 37 or at 39.5°C. As in cells undergoing autophagy, the cytoplasmic protein LC3B (LC3B-I) conjugates with phosphatidylethanolamine in the membrane of autophagosomes, and this leads to the appearance of LC3B+ vesicles that can be detected by confocal microscopy. Thus, the intensity of vesicular LC3B/cell serves as a readout of autophagy levels. Quantification of multiple images like those shown in Figure 2A, acquired from three independent experiments, indicated that a small but significant decrease in autophagy levels was detected in PMA-stimulated neutrophils at 39.5°C. These findings ruled out that an enhancement of autophagy is responsible for NETosis acceleration by hyperthermia (Figures 2B,C).

We then analyzed if STFRH modulates ROS production. As shown in Figure 2D, STFRH significantly increased PMA-induced ROS production, suggesting that this effect could contribute to the accelerated NETosis rates observed upon STFRH.

**Figure 3:**
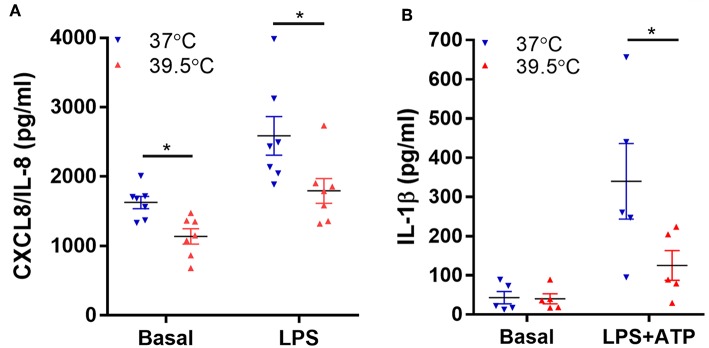
Short-term fever-range hyperthermia reduces pro-inflammatory cytokines secretion induced by LPS. Neutrophils were cultured for 1 h at 37 or 39.5°C in the absence (basal) or presence of LPS (250 ng/mL) and 2 h later **(B)** were stimulated with ATP (2.5 mM), and at 5 h post-LPS stimulation, cells were centrifuged, supernatants were collected, and the concentrations of CXCL8/IL-8 **(A)** and IL-1β **(B)** were determined by ELISA. Data depicted correspond to the mean value of cytokine concentrations secreted by 10^6^ neutrophils of assays performed in triplicate per donor. Bars represent the mean ± SEM of the independent experiments performed. *p < 0.01; two-way ANOVA with Bonferroni's multiple-comparisons test. LPS, lipopolysaccharide; CXCL8/IL-8, chemokine (C-X-C motif) ligand 8/interleukin 8; ELISA, enzyme-linked immunosorbent assay; ANOVA, analysis of variance.

Previous studies also showed that neutrophil exposure to 42°C for 1 h inhibits NF-κB activation induced by LPS as well as TNF-α mRNA expression, an effect that was also observed when neutrophils were exposed to 40°C (22). Thus, we then determined if a lower hyperthermic condition (39.5°C) is able to modulate neutrophil capacity to synthesize NF-κB-dependent pro-inflammatory cytokines (25). We evaluated both spontaneous and LPS-induced neutrophil CXCL8/IL-8 and IL-1β secretion after 1 h exposure to either 37 or 39.5°C and four additional hours at 37°C. For IL-1β determinations, ATP was added 2 h after LPS stimulation in order to induce the inflammasome activation and consequently IL-1β secretion (17). Our results indicated that STFRH significantly reduced both spontaneous and LPS-triggered CXCL8/IL-8 production (Figure 3A). By contrast, STFRH did not affect basal IL-1β release but significantly reduced LPS-induced IL-1β secretion (Figure 3B). Of mention, LPS is a weak NETosis inducer and, as we showed in Figure 1A, exerts anti-apoptotic effects that can be prevented by STFRH. Thus, to evaluate the potential contribution of a loss of viability to the effects induced by hyperthermia on cytokine production, we analyzed cell viability under each of the experimental conditions assessed. As shown in Figure S4, although STFRH significantly prevented the anti-apoptotic effect induced by LPS, the reduced magnitude of this effect at this time point suggests that the inhibition in cytokine secretion upon neutrophil febrile temperature exposure was not merely due to a differential cell death rate. This conjecture is supported by the fact that STFRH reduced CXCL8/IL-8 basal secretion (Figure 3A) even though it did not affect the viability of unstimulated neutrophils (Figure S4).

**Figure 4:**
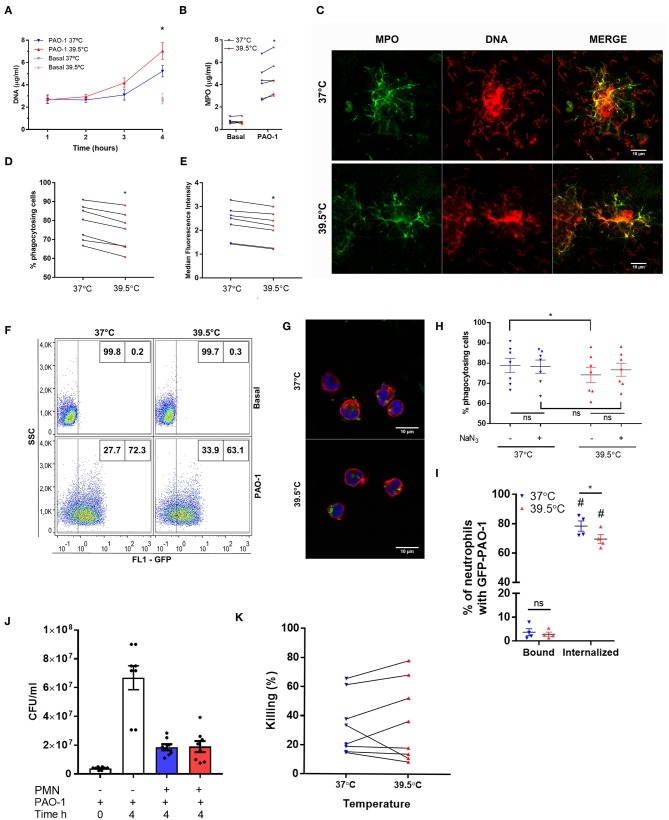
Short-term fever-range hyperthermia reduces bacterial phagocytosis and increases NET production induced by *Pseudomonas aeruginosa*. Neutrophils were cultured for 1 h at 37 or 39.5°C and then challenged with *P. aeruginosa* at MOI 1 **(A–C,J,K)** or MOI 10 **(D–I)** and cultured at 37°C for 1, 2, 3, or 4 h **(A)**, 4 h **(B,C,J,K)**, or 1 h (**D–I**; GFP-tagged *P. aeruginosa* PAO-1). After culture, **(A)** DNA and **(B)** MPO were evaluated as a readout of the presence of NETs. Data are depicted as the mean ± SEM of four **(A)** or six **(B)** independent experiments. *p < 0.01 39.5 vs. 37°C at 4 h. **(C)** cells were fixed and permeabilized, MPO was stained with a FITC-conjugated specific monoclonal antibody, and DNA was stained with PI. Representative confocal microscopy images of two experiments. **(D–F)** GFP-PAO-1 phagocytosis was evaluated by flow cytometry. **(D)** Percentage of GFP-positive cells (% of phagocytosing cells) and **(E)** GFP-median fluorescence intensity of neutrophils challenged with GFP-PAO-1; *p < 0.01 39.5 vs. 37°C. **(F)** Representatives dot plots of one of the experiments depicted in **(D)**. **(G)** Representative confocal microscopy images of neutrophils challenged with GFP-PAO-1 like in **(D)**, which at the end of the experiments were fixed and stained with TRITC–phalloidin (red) to delimitate cell contours, and TO-PRO-3 to stain DNA (blue). **(H)** Percentage of GFP-positive cells after treatment or not with NaN_3_ (100 μM) added 10 min before bacterial challenge; *p < 0.01, ns: non-significant. **(I)** After co-culture with GFP-PAO-1, cells were stained with an Alexa 647 anti-GFP antibody to discriminate extracellular (bound) bacteria from those internalized (unable to stain with anti-GFP antibody). #*p* < 0.0001 vs. bound at their respective temperatures; *p = 0.05, 39.5 vs. 37°C, ns: non-significant. **(J)** After co-culture, samples were centrifuged, and neutrophils were lysed and mixed with their respective supernatants previously treated with DNase to release bacteria entrapped in NETs and then were plated in LB agar. After culture for 20 h at 37°C, the CFUs were counted. PAO-1 time 0 h represents the original inoculum, and PAO-1 time 4 h represents CFU of bacteria cultured in the absence of neutrophils throughout the experiment. PAO-1 with neutrophils (PMN) previously cultured for 1 h at 37°C (blue bar) or 39.5°C (red bar). **(K)** Percentage of *P. aeruginosa* killing by neutrophils that were cultured at the indicated temperatures. *p < 0.01; two-way ANOVA with Bonferroni's multiple-comparisons test. NET, neutrophil extracellular trap; MOI, multiplicity of infection; GFP, green fluorescent protein; MPO, myeloperoxidase; FITC, fluorescein isothiocyanate; PI, propidium iodide; TRITC, tetramethylrhodamine; LB, Luria broth; CFUs, colony-forming units; PMA, phorbol myristate acetate; ANOVA, analysis of variance.

To determine if the impact of STFRH could be also observed when neutrophils were challenged with bacteria, we exposed neutrophils to either 37 or 39.5°C for 1 h and then co-incubated them with *P. aeruginosa* at MOI 1 for one, two, three, or four additional hours and then analyzed their ability to generate NETs. This experimental setting was chosen in order to avoid the contribution of the impact of hyperthermia on bacteria. As shown in Figures 4A,B, at 4 h p.s. STFRH significantly increased NETosis levels compared with those attained at 37°C, as indicated by increased concentrations of DNA and MPO detected in culture supernatants. Moreover, we confirmed that DNA detected in culture supernatants corresponded to NETs, as MPO was found colocalizing with DNA by confocal microscopy (Figure 4C). Noteworthy, bacteria were found ensnared in NETs.

We performed additional experiments to determine whether STFRH also modulates bacterial phagocytosis. To this end, we exposed neutrophils to either 37 or 39.5°C for 1 h and then co-incubated them with GFP-tagged *P. aeruginosa* for 1 h at 37°C, a time point at which NETs had not yet been detected in culture supernatants. As shown in Figures 4D–G, STFRH significantly reduced either the percentage of cells containing GFP-fluorescent bacteria or the median fluorescence intensity associated with neutrophils. However, considering that previous studies indicated that HOCl produced in the phagosome by the MPO–H_2_O_2_-chloride system can quench GFP fluorescence, we performed additional experiments in the presence of NaN_3_, a compound capable to inhibit this effect (19), to rule out a contribution of HOCl to the results obtained. Nevertheless, we did not find significant differences in the percentage of neutrophils with GFP fluorescence in the presence or absence of NaN_3_ at either 37 or 39.5°C (Figure 4H). Furthermore, we evaluated the percentage of GFP-fluorescent neutrophils, which, without being permeabilized, were also able to bind an anti-GFP antibody, as a readout of the cells with surface-attached bacteria (Figure 4I). Less than 5% of neutrophils exhibited GFP bacteria bound to the cell surface in contrast to ~70% that had ingested bacteria, but more importantly, no differences were found in the attached bacteria between both temperatures. Moreover, internalized bacteria could be visualized in a three-dimensional reconstruction from Z-stack images acquired by confocal microscopy of PAO-1-challenged neutrophils (Videos S2, S3). Collectively, these findings suggest that STFRH moderately reduces *P. aeruginosa* phagocytosis.

As STFRH inhibited *P. aeruginosa* phagocytosis but significantly increased NETosis, we then analyzed if STFRH modulates neutrophil microbicidal capacity against *P. aeruginosa*. To this end, we evaluated bacterial viability after co-culture for 4 h at 37°C with neutrophils that had been exposed for 1 h at either 37 or 39.5°C. As expected, co-culture with neutrophils markedly reduced the number of CFU than did bacteria that were cultured in the absence of neutrophils for 4 h (Figure 4J). However, STFRH did not significantly modulate neutrophil capacity to kill *P. aeruginosa* (Figures 4J,K), because when NETs were dismantled by DNase treatment, the number of CFU recovered from co-cultures of neutrophils subjected to each temperature was not significantly different. These findings suggest that hyperthermia might increase the capacity of neutrophils to wall off the infection but does not increase their microbicidal capacity, at least at this time point evaluated.

**Figure 5:**
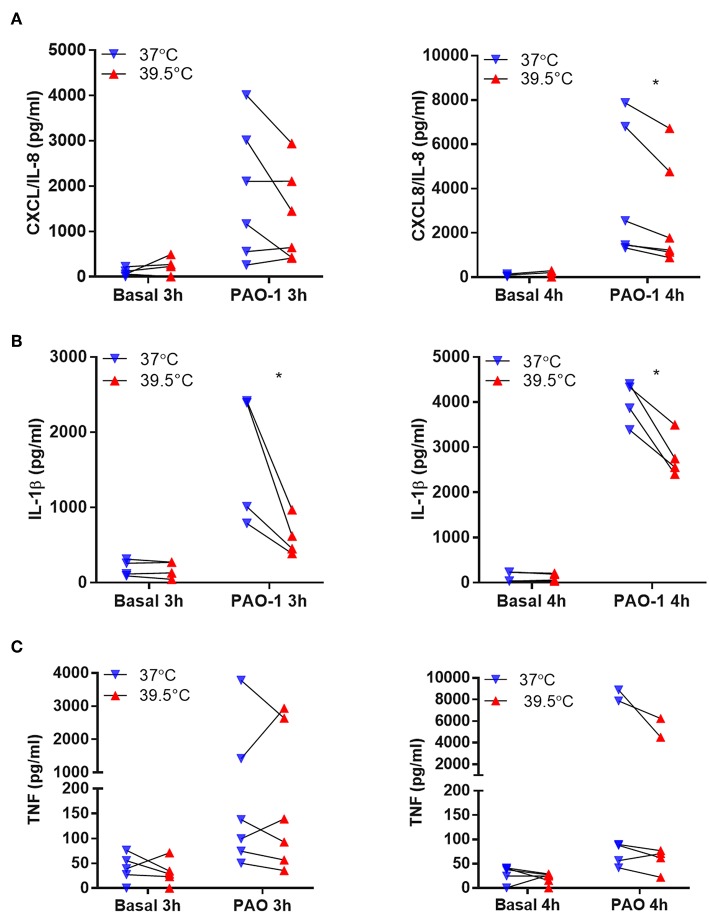
Short-term fever-range hyperthermia reduces the pro-inflammatory cytokines response to *Pseudomonas aeruginosa*. Neutrophils were cultured for 1 h at 37 or 39.5°C and then were challenged with *P. aeruginosa* MOI 0.1 for 3 or 4 h. Afterwards, cells were centrifuged, supernatants were collected, and the concentrations of CXCL8/IL-8 **(A)**, IL-1β **(B)**, and TNF-α **(C)** were determined by ELISA. Data depicted correspond to the mean value of cytokine concentrations secreted by 5 × 10^5^ neutrophils of assays performed in triplicate per donor. *p < 0.01; two-way ANOVA with Bonferroni's multiple-comparisons test. MOI, multiplicity of infection; IL, interleukin; TNF-α, tumor necrosis factor alpha; ELISA, enzyme-linked immunosorbent assay; ANOVA, analysis of variance.

In another set of experiments, we analyzed whether STFRH also modulates neutrophils to secrete cytokines when challenged with bacteria. Thus, to avoid a potential contribution of a loss of viability to cytokine extracellular concentrations, we evaluated neutrophil response to *P. aeruginosa* at a lower MOI (0.1). To this end, we exposed neutrophils to either 37 or 39.5°C for 1 h and then challenged them with *P. aeruginosa* for three or four additional hours. Then, we determined the concentrations of CXCL8/IL-8, IL-1β, and TNF-α in culture supernatants. Results indicated that hyperthermia significantly reduced the secretion induced by *P. aeruginosa* of CXCL8/IL-8 at 4 h of culture and IL-1β at either 3 or 4 h of culture (Figures 5A,B). By contrast, TNF-α secretion was not significantly modulated by hyperthermia (Figure 5C). In order to rule out that the reduction in cytokine secretion observed was due to differential cell death, we also evaluated neutrophil viability in the pellets of the same samples by analyzing the number of cells that had lost plasma membrane integrity by Vivafix staining and flow cytometry. We did not detect significant differences in the percentages of dead cells at both temperatures when evaluated at either 3 or 4 h of culture (Figure S5). We obtained similar results when we evaluated cell viability by annexin V-FITC and PI staining (Figure S6A) and lytic cell death by analyzing the presence of LDH in cell supernatants (Figure S6B), even though *P. aeruginosa* was still able to induce some NETosis at this low MOI (0.1) (Figure S7). Altogether, these results indicated that STFRH reduces pro-inflammatory cytokine secretion upon challenge with *P. aeruginosa* and this effect is not associated with a decreased cell viability.

## Discussion

In this study, we determined that STFRH accelerates NETosis. Our findings indicated that PMA-induced NETs generation was increased when cells were exposed to hyperthermia for only 1 h. Moreover, we observed that ROS production triggered by PMA was also increased by STFRH, suggesting that this effect could contribute to the accelerated NETosis rates. By contrast, autophagy levels induced by PMA were not increased by STFRH, ruling out a causal relationship with NETosis acceleration. We also determined that STFRH accelerated NETosis induced by *P. aeruginosa* but reduced the capacity of neutrophils to phagocytose the bacteria. However, STFRH did not affect the neutrophil capacity to kill *P. aeruginosa* after 4 h of co-culture, suggesting that hyperthermia might contribute to bacterial ensnarement to avoid its dissemination.

Studies performed by Kettritz et al. indicated that short exposure (1 h) of neutrophils to 42°C modulated neither their spontaneous apoptosis nor the anti-apoptotic effect of GM-CSF, IL-8, and dexamethasone but prevented the anti-apoptotic effect triggered by LPS evaluated 20 h later (22). However, the authors showed that a short exposure of neutrophils to either 39 or 40°C did not modulate their apoptosis levels after 20 h of being left unstimulated and, at this time point, slightly counteracted the LPS anti-apoptotic effects only upon short exposure at 40°C. In this study, we confirmed that STFRH does not modulate neutrophil spontaneous apoptosis and, in line with Kettritz et al.'s findings, determined that the ability of STFRH to prevent the LPS anti-apoptotic effect is already evident at early stages. Other studies showed that 90 min at 39 or 41°C inhibited the spontaneous apoptotic DNA fragmentation after 24 h of culture and preserved the membrane asymmetry characteristic of viable cells, even though it surprisingly decreased CD16 expression (26). Whether this hyperthermia-triggered behavior of unstimulated cells increases their propensity to undergo NETosis upon agonist stimulation remains to be determined. Other studies demonstrated that long-term FRH (8 h or more) accelerates caspase-dependent apoptosis (21), in contrast to the effects exerted by short-term exposure to fever-range temperatures. Thus, it appears that both the time extent to FRH and the temperature to which cells are exposed differentially modulate either spontaneous or induced cell death.

On the other hand, in studies addressing the effects of anti-inflammatory drugs on NET formation, similar to what we found here after 1 h of FRH, the authors showed an increased NETs release after 3 h of exposure of neutrophils at 40°C in the presence of PMA. However, in this study, only an ELISA to detect nucleosomes was employed as a readout of the presence of NETs (27).

We also found that in contrast to the impact on NET production, STFRH reduced *P. aeruginosa* phagocytosis by neutrophils. Moreover, our findings indicated that although neutrophils were able to kill *P. aeruginosa*, STFRH did not modulate this function, at least after 4 h of co-culture. However, we cannot rule out that longer exposure of bacteria to the network represented by NETs, which were increased by STFRH, can lead to differential killing at later time points. Our results let us speculate that the reduced capacity to kill the bacteria by phagocytosis observed upon STFRH conditions is counterbalanced by an increased NETosis.

Our studies with the caspase-1 inhibitor Ac-YVAD-cmk indicated that STFRH promotes the canonical NETosis mechanism that is a caspase-1-independent process (5). Recent studies indicated that *P. aeruginosa* PAO-1 after engulfment by macrophages is able to escape the phagosome to the cytosol (28). *Citrobacter rodentium* (a Gram-negative bacterium that is well-established to escape the phagosome) was shown to induce caspase-4-dependent NETosis of human neutrophils by non-canonical inflammasome activation (29). In the light of these new findings, we cannot rule out that STFRH is also enhancing NETosis that might be induced by PAO-1 through this pathway. Further studies are required to address this possibility.

Kettritz et al. also found that short neutrophil exposure (1 h) to 40°C inhibited NF-κB activation as it decreased LPS-induced IκBα degradation and IκBα mRNA expression (22). Results of our study indicating that STFRH inhibits the secretion of CXCL8/IL-8 and IL-1β, two cytokines that are synthesized in an NF-κB-dependent manner (25), are in line with these findings. These authors also determined that a short neutrophil exposure (1 h) to 40°C reduced TNF-α mRNA expression induced by LPS. However, in our study, STFRH did not significantly modulate neutrophil TNF-α secretion induced by *P. aeruginosa*, although a reduction in this cytokine secretion was observed in five out of six donors analyzed after 4 h of PAO-1 challenge.

Interestingly, in a mice model of experimental peritonitis induced by *Klebsiella pneumoniae*, FRH reduced by 100,000-fold the intraperitoneal bacterial burden and increased mice survival from 0 to 50% in comparison with that in normothermic infected animals (30). The authors showed that FRH did not affect the bacterial *in vitro* proliferation rates, which suggested that enhanced host defense mechanisms accounted for the reduction in pathogen load. However, in another study aimed to analyze FRH impact on pneumonia caused by the same pathogen, although FRH (core temperature ~39°C) diminished in 400-fold intrapulmonary bacterial burden, unlike the peritonitis model, it conferred no survival advantage (11). Moreover, the authors found that hyperthermia also accounted for a marked increase in mortality either in the *K. pneumoniae*-induced pneumonia treated with antibiotics or in the intra-tracheal LPS challenge, which were both non-lethal in euthermic mice (11). Altogether, these findings lead the authors to suggest that the host response in the hyperthermic mice rather than the pathogens themselves might contribute to death. These findings suggested that differences in mice survival in the peritonitis and pneumonia models under hyperthermic conditions were a consequence of a balance between an expedited bacterial clearance and increased collateral tissue damage (31). In line with this possibility, FRH greatly accelerated lethal lung injury in a mice model of non-infectious injury caused by oxygen toxicity (12). Noteworthy, an increased neutrophil infiltration upon exposure to FRH was observed in both LPS and hyperoxia-induced acute lung injury models (11, 12). In previous studies, we demonstrated that NETs promote a pro-inflammatory response by stimulating the production of higher levels of CXCL8/IL-8 and IL-6 by airway epithelial cells and macrophages (18). Thus, regardless of the beneficial antimicrobial properties of NETs, their exacerbated production by STFRH might contribute to increased inflammation in response to Gram-negative bacterial infection.

Studies performed by Schauer et al. showed that neutrophil stimulation with monosodium urate crystals at high cellular density induces the formation of aggregated NETs structures, which proteolytically degrade cytokines and chemokines produced by mouse neutrophils and consequently reduce inflammation (32). Thus, it is tempting to speculate that the increased NET production promoted by STFRH might also contribute to the reduced cytokine levels observed under hyperthermic conditions. If this were the case, it might represent an additional mechanism to restrain excessive inflammation.

Studies performed by Bzowska et al. described that exposure of human neutrophils to STFRH in the absence of stimulation results in a non-phlogistic recognition by macrophages (26). Thus, additional mechanisms might also act in concert to lessen inflammation incited by hyperthermia not only by amplifying NET production but also by acting on other cell types (11, 31).

Collectively, the results of our study suggest that fever spikes could lead neutrophils to trigger an emergency response promoting NET formation to ensnare bacteria in order to wall off the infection. However, taking into account the pro-inflammatory properties ascribed to NETs (18, 33–35), we speculate that the ability of STFRH to prevent the LPS-induced anti-apoptotic effect together with its capacity to reduce neutrophil pro-inflammatory cytokine secretion might represent feedback mechanisms to limit an increased inflammatory response that might be detrimental for the host (Figure S8).

## Data Availability Statement

The datasets generated for this study are available on request to the corresponding author.

## Ethics Statement

The studies involving human participants were reviewed and approved by Ethical Committee of the Institutos de la Academia Nacional de Medicina. The participants provided their written informed consent to participate in this study.

## Author Contributions

IK and FS designed the experiments, carried out most of them, analyzed and interpreted the data. CS, DR, MM, and MG conducted some experiments. FF programmed the image quantification macro, and performed microscopy acquisitions together with IK and FS. CG and CL contributed to bacterial assays. JG, JRG, and CJ provided scientific expertise and contributed analysis and data interpretation. MIG and AT designed experiments and analyzed and interpreted the data. AT conceived the research and wrote the manuscript. All authors reviewed the manuscript.

### Conflict of Interest

The authors declare that the research was conducted in the absence of any commercial or financial relationships that could be construed as a potential conflict of interest.
