# Supplementary material for: Short-Term Fever-Range Hyperthermia Accelerates NETosis and Reduces Pro-inflammatory Cytokine Secretion by Human Neutrophils
Source: Front Immunol. 2019 Oct 18;10:2374. doi: 10.3389/fimmu.2019.02374 (PMC6813732; doi:10.3389/fimmu.2019.02374)
Supplement: Supplementary file 1 [file Data_Sheet_1.pdf]

Legends to videos

Video 1. NETosis induced by PMA in neutrophils subjected to STFRH.

Neutrophils ($4x10^5$) were stimulated in a humidified atmosphere containing 5% CO_2 with PMA (25 ng/ml), and were incubated for 1 h at 39.5°C and then at 37°C. Along this time neutrophils were monitored by live cell imaging in three different channels: differential interface contrast (DIC), the cell permeable DNA dye NucSpot live 488 (green) and the cell impermeable DNA dye propidium iodide (red). Cells were monitored every 30 seconds for 150 min.

Video 2. GFP-tagged *P. aeruginosa* PAO-1 phagocytosis at 37°C.

Neutrophils ($1x10^6$) were cultured for 1 h at 37°C, and then challenged with GFP-tagged *P. aeruginosa* (green) at MOI 10. At the end of the experiment, cells were fixed and stained with TRITC-Phalloidin (red) to delimitate cell contours, and TO-PRO-3 to stain DNA (blue). Then, samples were cytospinned, mounted with Aqua-Poly/Mount mounting medium and examined by CLSM. For 3D reconstructions, confocal microscopy images were acquired in slices taken at 800 nm intervals in the z dimension. Reconstructions were made using the 3D viewer plugin in the Fiji software displaying the z-stack in the volume mode with a resampling factor of 2. Finally, a video was made manually rotating the reconstructed structures. Results are representative of at least 4 independent experiments.

Video 3. GFP-tagged *P. aeruginosa* PAO-1 phagocytosis at 39.5°C.

Neutrophils ($1x10^6$) were cultured for 1 h at 39.5°C, and then challenged with GFP-tagged *P. aeruginosa* (green) at MOI 10. At the end of the experiment cells were fixed and stained with TRITC-Phalloidin (red) to delimitate cell contours, and TO-PRO-3 to stain DNA (blue). Then, samples were cytospinned, mounted with Aqua-Poly/Mount mounting medium and examined by CLSM. For 3D reconstructions, confocal microscopy images were acquired in slices taken at 800 nm intervals in the z dimension. Reconstructions were made using the 3D viewer plugin in the Fiji software displaying the z-stack in the volume mode with a resampling factor of 2. Finally, a video was made manually rotating the reconstructed structures. Results are representative of at least 4 independent experiments.
