# Supplementary material for: Short-Term Fever-Range Hyperthermia Accelerates NETosis and Reduces Pro-inflammatory Cytokine Secretion by Human Neutrophils
Source: Front Immunol. 2019 Oct 18;10:2374. doi: 10.3389/fimmu.2019.02374 (PMC6813732; doi:10.3389/fimmu.2019.02374)
Supplement: Supplementary file 2 [file Data_Sheet_2.PDF]

Supplementary material

Figure S1

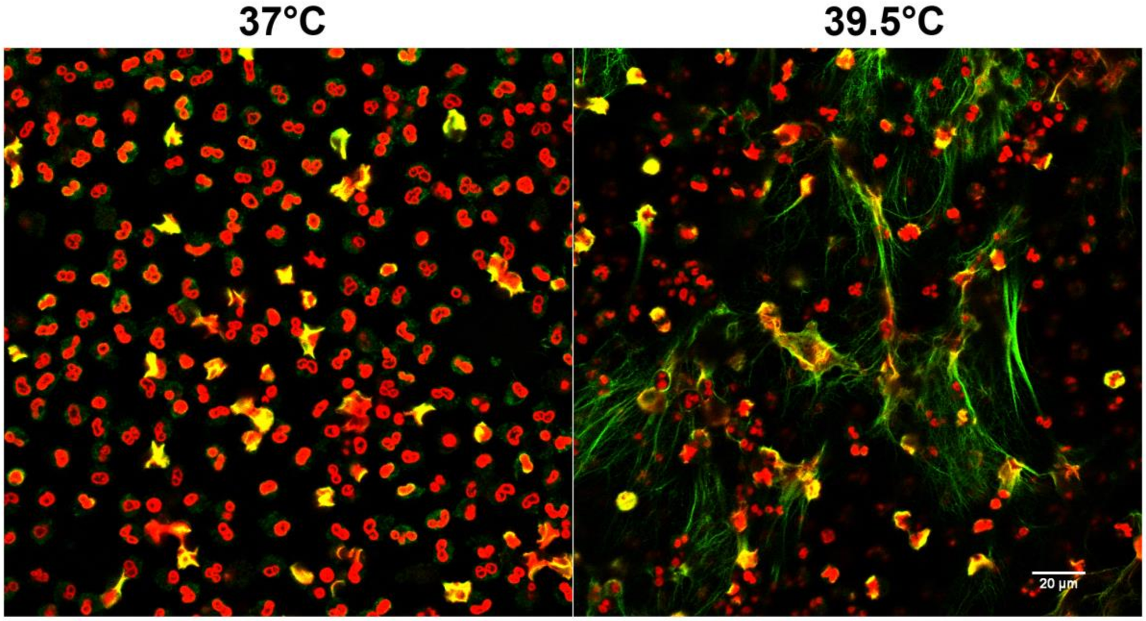

Neutrophils were cultured for 1 h at 37°C or 39.5°C in the presence of PMA (100 nM) and then cultured at 37°C for one additional hour in Lab-tek chambers. Then, cells were fixed with PFA 4%, permeabilized with acetone and immunostained with anti-MPO (green) and stained with propidium iodide (1 µg/ml; red). Images were acquired by confocal microscopy with a Plapon 60X/NA1.42 objective.

Figure S2

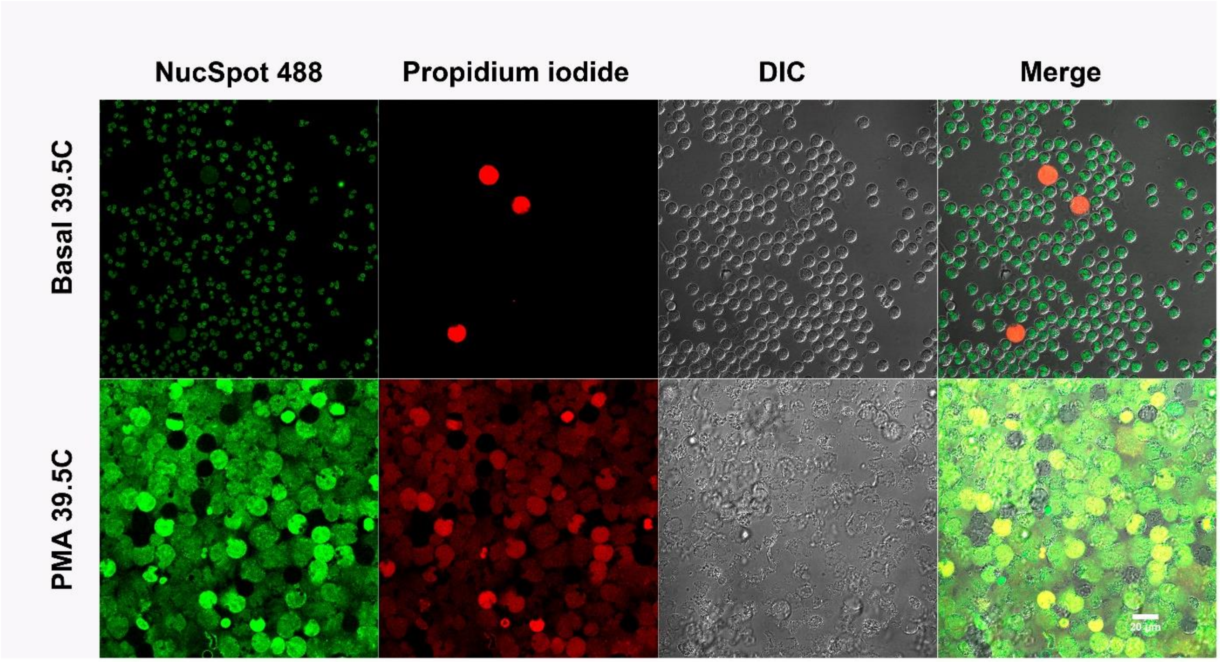

Neutrophils were cultured on poly-L-lysine-coated Cellview glass bottom dishes (Greiner Bio One) inside a temperature-controlled chamber under a humidified atmosphere with 5% CO_2. with NucSpot 488 (Biotium), a cell-permeable DNA-dye, and propidium iodide (which only penetrates in cells with a compromised integrity of their plasma membranes), for 1 h at 39.5°C in the absence (upper images) or presence (bottom images) of PMA (25 ng/ml). Then, were cultured at 37°C for 2 h 40 min and imaged by confocal microscopy. Images were acquired by a confocal microscope with a Plapon 60X/NA1.42 objective.

Figure S3.

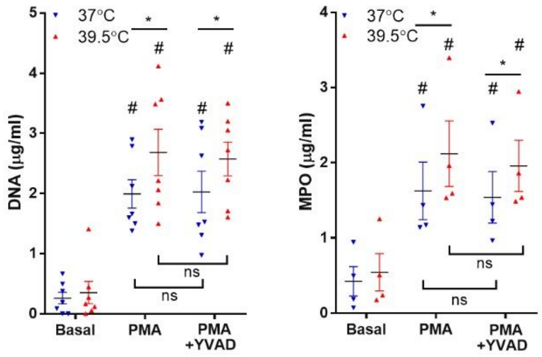

Caspase-1 is not involved in NETosis promotion by STFRH. Neutrophils were cultured for 20 min at 37°C in the absence or presence of the caspase-1 inhibitor Ac-YVAD-cmk (YVAD; 50 µM) and then for 1 h at 37°C or 39.5°C in the absence (basal) or presence of PMA (25 ng/ml). Then, cells were cultured at 37°C for three additional hours and DNA (left) and MPO (right)

concentrations in culture supernatants were determined. Bars represent the mean ± SEM of the independent experiments depicted. Each data depicted represents the mean value of assays performed in duplicate per donor. *p<0.01 PMA 37°C vs PMA 39.5°C and PMA+YVAD 37°C vs PMA+YVAD 39.5°C; #p<0.01 PMA or PMA+YVAD vs basal at their respective temperatures; Two-way ANOVA with Bonferroni´s multiple comparisons test.

Figure S4.

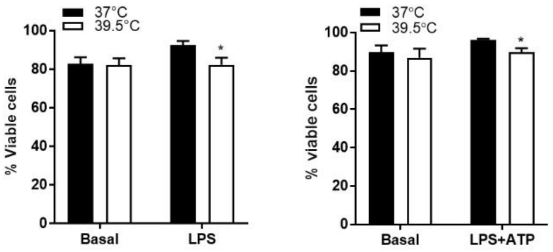

Neutrophil viability evaluated by annexin V/PI staining and flow cytometry. Neutrophils were cultured for 1 h at 37°C or 39.5°C in the absence (basal) or presence of LPS (250 ng/ml) and then cultured at 37°C for four additional hours. Then, neutrophil viability was evaluated by annexin-V FITC/propidium iodide staining and flow cytometry. Data are depicted as the mean ± SEM of viable cells of 6 (LPS) and 4 (LPS+ATP) experiments. *p<0.05; Two-way ANOVA with Bonferroni´s multiple comparisons test.

Figure S5.

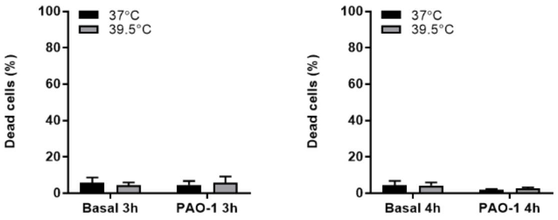

Neutrophil were incubated at either 37°C or 39.5°C for 1 h and then were challenged with *P. aeruginosa* PAO-1 at MOI 0.1 for 3 (left graph) or 4 h (right graph) additional hours. Then, the percentage of cells with bright VivaFix fluorescence was determined by flow cytometry. Data represent the mean ± SEM of 4 experiments.

Figure S6.

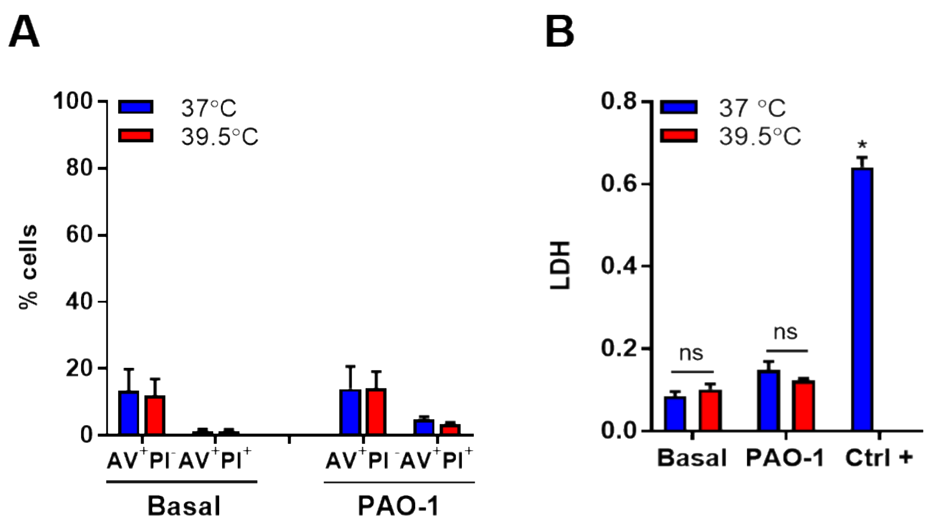

Neutrophil were incubated at either 37°C or 39.5°C for 1 h and then were challenged with *P. aeruginosa* PAO-1 at MOI 0.1 for 4 additional hours at 37°C. Then, in (A) viability was evaluated in cell pellets by annexin-V FITC/propidium iodide (AV PI) staining and flow cytometry; and in (B) LDH was determined in culture supernatants. Ctrl+ corresponds to LDH levels of lysed neutrophils with the same number of cells employed in the experiments. Data are depicted as the mean ± SEM of 4 (A) and (5) experiments. Differences between neutrophils challenged with PAO-1 at both temperatures were non-significant. Two-way ANOVA with Bonferroni´s multiple comparisons test.

Figure S7.

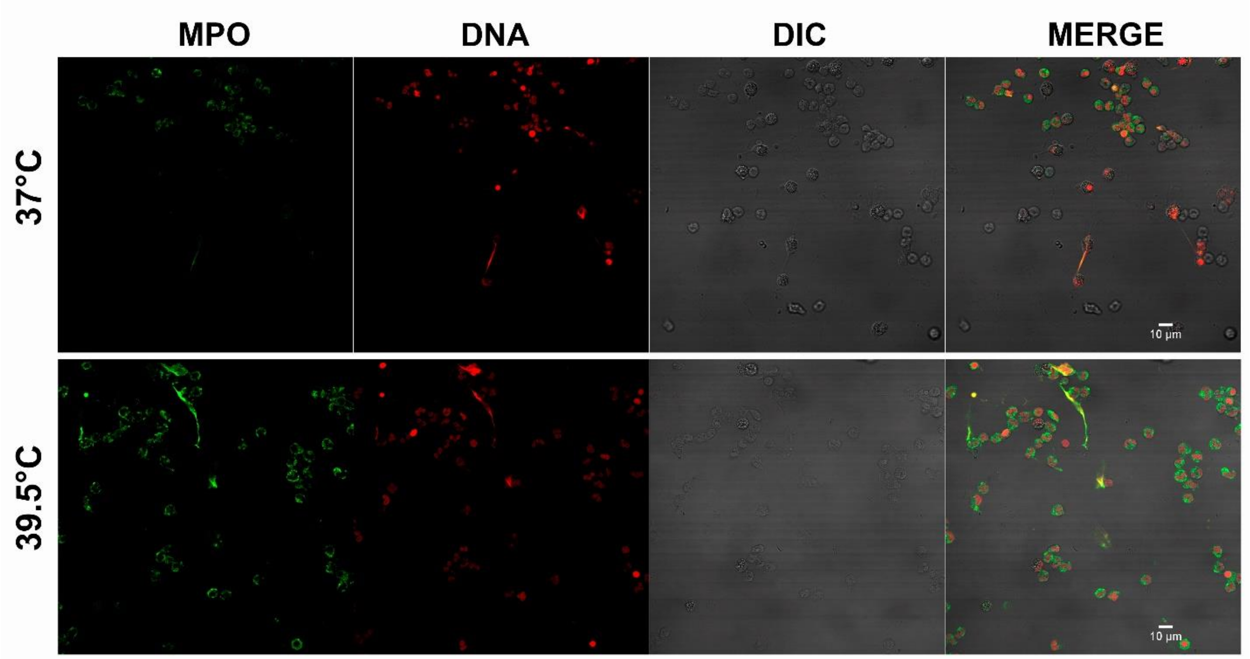

Neutrophils were cultured for 1 h at 37°C or 39.5°C, and then challenged with *P. aeruginosa* MOI 0.1 and cultured at 37°C for 4 additional hours. After culture, cells were fixed, permeabilized, MPO was stained with a FITC-conjugated specific monoclonal antibody and DNA was stained with PI. Representative confocal microscopy images of 2 experiments.

Figure S8.

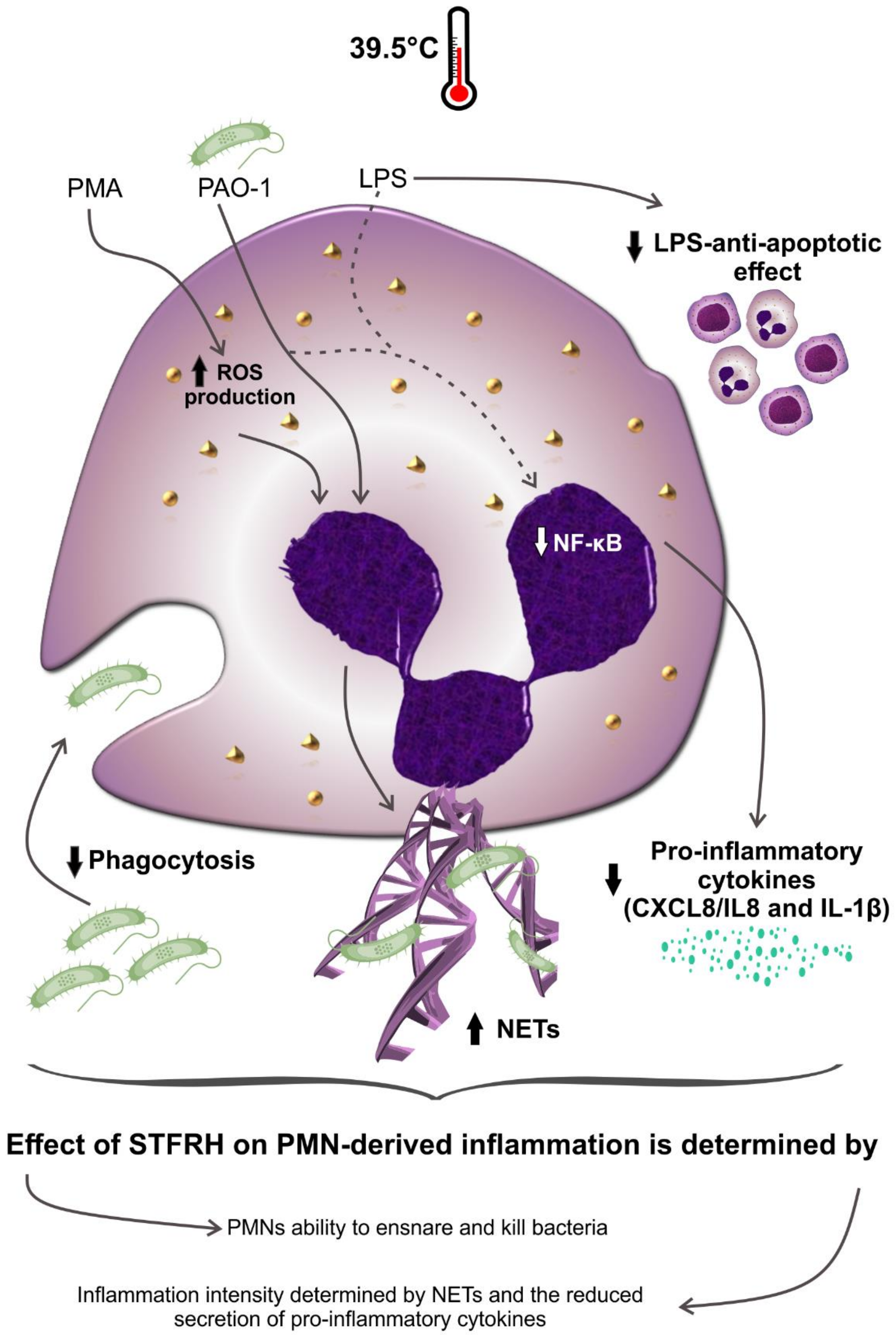

Model describing the outcome of short hyperthermia on inflammation by modulating neutrophil functions upon bacterial infections.
